# An investigation of emotion dynamics in major depressive disorder patients and healthy persons using sparse longitudinal networks

**DOI:** 10.1371/journal.pone.0178586

**Published:** 2017-06-01

**Authors:** Stijn de Vos, Klaas J. Wardenaar, Elisabeth H. Bos, Ernst C. Wit, Mara E. J. Bouwmans, Peter de Jonge

**Affiliations:** 1 University of Groningen, University Medical Center Groningen, Interdisciplinary Center Psychopathology and Emotion regulation, Groningen, the Netherlands; 2 University of Groningen, Faculty of Behavioural and Social Sciences, Department of Developmental Psychology, Groningen, the Netherlands; 3 University of Groningen, Johann Bernoulli Institute of Mathematics and Computer Science, Groningen, the Netherlands; University of Amsterdam, NETHERLANDS

## Abstract

**Background:**

Differences in within-person emotion dynamics may be an important source of heterogeneity in depression. To investigate these dynamics, researchers have previously combined multilevel regression analyses with network representations. However, sparse network methods, specifically developed for longitudinal network analyses, have not been applied. Therefore, this study used this approach to investigate population-level and individual-level emotion dynamics in healthy and depressed persons and compared this method with the multilevel approach.

**Methods:**

Time-series data were collected in pair-matched healthy persons and major depressive disorder (MDD) patients (n = 54). Seven positive affect (PA) and seven negative affect (NA) items were administered electronically at 90 times (30 days; thrice per day). The population-level (healthy vs. MDD) and individual-level time series were analyzed using a sparse longitudinal network model based on vector autoregression. The population-level model was also estimated with a multilevel approach. Effects of different preprocessing steps were evaluated as well. The characteristics of the longitudinal networks were investigated to gain insight into the emotion dynamics.

**Results:**

In the population-level networks, longitudinal network connectivity was strongest in the healthy group, with nodes showing more and stronger longitudinal associations with each other. Individually estimated networks varied strongly across individuals. Individual variations in network connectivity were unrelated to baseline characteristics (depression status, neuroticism, severity). A multilevel approach applied to the same data showed higher connectivity in the MDD group, which seemed partly related to the preprocessing approach.

**Conclusions:**

The sparse network approach can be useful for the estimation of networks with multiple nodes, where overparameterization is an issue, and for individual-level networks. However, its current inability to model random effects makes it less useful as a population-level approach in case of large heterogeneity. Different preprocessing strategies appeared to strongly influence the results, complicating inferences about network density.

## Introduction

Although major depressive disorder (MDD) is predicted to become one of the most important contributors to the global burden of disease [1], the condition remains poorly understood. Despite many research efforts, the etiological mechanisms underlying depression are still unclear and accurate prediction of outcome and course in MDD patients has proven difficult. An important reason for this may lie in the heterogeneity of MDD, both in terms of the possible variations in patients’ symptom patterns and in course trajectories [2,3]. Furthermore, the definition of MDD as formulated in the Diagnostic and Statistical Manual (DSM) is based on clinical consensus rather than empirical research, limiting its validity and usefulness in scientific research [4, 5].

Researchers have attempted to develop alternative, data-driven diagnostic systems focusing either on the identification of symptom dimensions or discrete sub/prototypes to provide more homogeneous descriptions of psychopathology, in particular MDD [6]. Most researchers have used latent variable models (LVM) to identify such symptom dimensions (e.g. [7, 8]) or subtypes (e.g. [9, 10]). However, it has been argued that, like DSM classifications, the diagnostic entities resulting from LVMs do insufficient justice to the dynamic nature of psychopathology because they assume that all observed symptoms are manifestations of a single underlying disorder or severity construct (e.g. [11]). Because of this, these diagnostic descriptions provide no insight into temporal relationships and interactions between individual symptoms. This is unfortunate, because in real life symptoms may develop consecutively, with the onset of one symptom influencing the onset and/or persistence of other symptoms [12]. It has been suggested that such dynamic processes could be captured using techniques from network analysis, which could provide deeper insights into the dynamic phenomenology of psychopathology and the underlying mechanisms (e.g. [11, 12, 13]).

In the network framework, psychopathology is represented as a network, in which nodes that represent symptoms or emotions are connected to each other by edges that represent some measure of association (e.g. correlations or regression coefficients). This approach can be used to visualize and analyze the relationships between responses on questionnaires or symptoms reported in clinical interviews. For instance, a simple matrix of (partial) correlations between symptoms can be visualized as a network, enabling subsequent analyses with dedicated techniques from graphical modeling/network analysis (e.g. centrality indices, connectivity indices, clustering algorithms). Network analyses can both be used to investigate the cross-sectional correlations between symptoms at a specific point in time and to investigate temporal associations between symptoms or emotions in longitudinal studies. Most studies have thus far used the former approach to get some insight into the cross-sectional correlation networks and, for instance, the centrality or clustering of certain symptoms within these networks (e.g. [11, 14]).

Interestingly, the longitudinal network approach, which allows for the investigation of how symptoms or emotions influence each other over time, has been used less often, although a few studies have used networks to analyze the structure of longitudinal associations in a multilevel data analysis framework [15–18]. The relative scarcity of longitudinal network models may lie in the associated analytical difficulties. A key challenge lies in distinguishing the relevant, longitudinal within-person correlations between emotions over time from the contemporaneous correlations at each time point. In addition, the question has remained unanswered whether longitudinal emotion networks are best studied at the population level or by generating a separate network for each individual member of the population. This latter approach might be optimal to gain insight into the actual temporal dynamics between emotions as they occur in individuals, because it would prevent the observed effects from being obscured by averaging across a heterogeneous pool of individuals. When using large numbers of repeated measures, the data of a single individual can be treated as a time series, allowing for data analysis at the person level and generation of person-specific networks. However, estimation of such person-specific longitudinal networks is not a trivial matter.

Fortunately, analytical methods have recently been developed that are specifically aimed at analyzing individual time-series data using network techniques. Abegaz and Wit [19] introduced a method that uses a penalized vector autoregression (VAR) model to estimate both contemporaneous and temporal associations in time-series data and demonstrated the use of this method in gene expression data. Using this approach, the contemporaneous and temporal associations can be investigated separately. In addition, the penalized approach has the advantage of reducing the number of spurious associations included in each of the networks.

The present study was aimed to apply the sparse longitudinal network approach to time series of affective states (further called ‘emotions’) (90 measurements; 3 times per day), collected in a group of 27 MDD patients and a group of 27 pair-matched healthy subjects. Both population-level and individual-level analyses were performed. First, network analyses were performed on the pooled time-series data of the MDD group and on those of the healthy group. The resulting networks were compared in terms of overall connectivity and the nodes with the largest in- and out-strength. To investigate how the sparse VAR results compare to results obtained with the previously used multilevel approach [15–18], the population-level networks were also estimated with the multilevel approach and compared to the sparse VAR-based networks. In addition, because different data pre-processing steps can be used (i.e. detrending or not; transformation or not; group-wise vs. individual-wise preprocessing), the effects of different preprocessing choices were evaluated. Next, network analyses were conducted on each subject’s individual data, and it was evaluated whether the connectivity of individual networks was associated with depression status (MDD vs. healthy) and other clinical and personality factors, in order to gain insight into the possible determinants of inter-individual differences in emotion dynamics. In line with the network perspective of psychopathology as a dynamic network of interacting symptoms [13, 16], this study focused on the temporal associations.

## Materials and methods

### Participants and procedures

Data were collected as part of the Mood and Movement in Daily Life (MOOVD) study [20]. The study included 27 MDD patients and 27 healthy persons, pair-matched by age, gender, smoking behavior, and BMI. During a period of 30 consecutive days, participants filled in a questionnaire thrice per day using an electronic diary. This procedure led to a dataset with up to 90 repeated assessments per subject. The study protocol was approved by the Medical Ethical Committee of the University Medical Center Groningen and conducted in accordance with the Declaration of Helsinki. All participants provided written informed consent.

### Measures

A list of self-report items was administered at each time point with an electronic device (PsyMate, PsyMate BV, Maastricht, the Netherlands). Seven positive affect (PA) items (feeling talkative, enthusiastic, confident, cheerful, energetic, satisfied, and happy) and seven negative affect (NA) items (feeling tense, anxious, distracted, restless, irritated, depressed, and guilty) were used. These items were adapted from a previous study [21] where they had proven to be very useful for experience sampling research. The items were rated on a 7-point Likert response scale. The affect items are further called ‘emotions’, in line with previous studies on this topic (e.g. [16, 17]). At baseline, the BDI [22] was used to assess depression severity in the MDD and control group. A person was placed in the control group if the BDI score was less than 9 and placed in the MDD group if the BDI score was over 14. Next, the Composite International Diagnostic Interview (CIDI [23]) was administered to verify the presence (MDD group) or absence (control group) of a current or recent (<2 months) diagnosis of MDD according to DSM-IV criteria. The Eysenck Personality Questionnaire [24] was used to assess baseline neuroticism in all subjects.

### Statistical analyses

#### Data preprocessing

The percentage of missing observations was 8.2% and 6.8% in the MDD and control group, respectively. Length of the time series was 83.2 on average (sd = 7.4). Missing data points were imputed with multiple imputation using R-package ‘Amelia’ [25]. The length of the time series after imputation was 90. All analyses were performed in ten imputed datasets and results were subsequently averaged. Because VAR models assume stationary data, we detrended the data by (i) fitting a nonparametric smoothing spline to the univariate time series of each item (using R’s *smooth*.*spline* function, choosing degree of freedom = 2), and by (ii) subtracting this curve from the original time series. Since the data consisted of Likert-style responses with skewed distributions, we relaxed the assumption of normality by transforming each item using the normal quantile transformation described in [26]. This transformation is similar to a Gaussian copula in the sense that the marginals are non-parametrically estimated empirical cumulative density functions; see also [27]. Both preprocessing steps (detrending and quantile transformation) were done per item and pooled over all individuals of the group for the population-level analyses; and per item per individual for the individual-level analyses. Other preprocessing steps have been previously used in longitudinal network studies. For instance, Bringmann et al. [16] used person-mean centering without detrending and did not transform the data. To investigate the effect of our preprocessing choices on the network results, the multilevel analyses were run multiple times with different sets of preprocessing steps.

#### Network estimation

A modified VAR model was used for the current analyses [19]. This model is different from a standard VAR model in that the estimation of the contemporaneous and temporal associations is performed using a regularized estimation approach [28]. In a standard approach, the parameters of a model may be estimated by maximizing the (log-)likelihood of the model. However, with a regularization approach this likelihood is modified to include a penalty function, which grows with the number and/or size of the model parameters, resulting in a sparser model compared to the model that would be estimated with a standard likelihood. In some situations, regularization allows one to estimate a model when there are more variables than sample points (e.g. in genetic datasets), but more generally, facilitates the interpretability of model results. Because the population-level networks were devoid of edges when using an information-criterion-type selection method, the regularization hyper-parameters λ_1_ and λ_2_ were set to 0.38 and 0.05, respectively, for all network analyses. These values were chosen because the regularization values provided by the default optimality criterion resulted in a network structure that was nearly empty and not useful for our purposes. Instead, we picked a value for λ_1_ from a range of possible values by refitting the population level models to 100 simulated dataset using a block-bootstrap and selecting a value for λ_1_ from the interquartile range. In addition, we set λ_2_ at 0.05, to achieve a suitable sparsity. See [28] for more on the role of hyper-parameters in regularization.

The connection between VAR models and networks has been described previously [29, 30]. When a VAR model is operationalized as a network model, the regression coefficients correspond to the edge weights in the network. If *A* represents the matrix of regression coefficients between the outcomes at time *t* versus the outcomes at time *t-1*, then the entry *a* of *A* at place (*j*,*k*) represents the association between item *j* at time *t-1* and item *k* at time *t*. Because the regression coefficients in a VAR model represent relations over time, the edges are arrows instead of lines, giving a directed (longitudinal) network. The other main parameters of the VAR model, i.e. the covariance matrix of the noise at time *t* representing the contemporaneous associations can also be visualized in a network. The entries of this matrix represent the estimated covariance between item *j* and *k*. These can be converted into a partial correlation matrix and visualized in an undirected contemporaneous network. To gain insight into the group- and individual differences in emotion dynamics over time, the main focus of this study was on the regression coefficients and the associated directed network. In addition, the covariance matrix was estimated and the associated undirected network inspected. To calculate the partial correlation matrix, the inverse of the covariance matrix was taken, resulting in the so called *precision matrix* (see [19]), which was rescaled to a partial correlation matrix [31]. All VAR models were run with time lag 1. In the population-level analysis, data from persons are treated as replications from the same population (i.e. the model is a constant coefficient model [32]).

In addition to the sparse VAR-based models, population-level networks were also estimated with the multilevel approach presented by Bringmann et al. [16]. In this multilevel approach, univariate multilevel regression models are fitted to each emotion variable, using the lagged values of all other emotion variables as predictors. The models were estimated using the lme function of the R package *nlme*. Emotion scores were person-mean centered in advance. Random slopes were included for each variable in the model. The need to include a time trend was tested by comparing models with and without time trend (average BIC across univariate models). Models without time trend were favored by the BIC comparisons. Mimicking the Bringmann et al. [16] code was not completely possible, as having 14 items meant that we had to specify a parsimonious random effect covariance structure to get the *lme* function to run at all (independent vs. unstructured covariance structure).

All above described analyses were run treating the overnight lag similarly to the other time lags in the dataset. By using this approach, one assumes that the overnight lag is comparable to the in-day time lags (e.g. morning-afternoon), which may not be the case. Therefore, we also reran the multilevel analyses with the overnight lag set to missing, to investigate the effect of including vs. excluding the overnight lag. This was only done with the multilevel approach because this approach has been most widely used to previously analyze diary data and is capable of handling missing data points.

#### Analyses of the population networks

After network estimation in the healthy and MDD groups, the resulting networks were compared on several aspects. First, incoming and outgoing edges were investigated by looking at the in- and out-strength of each node. The in- and out-strength of a node is computed by summing the absolute values of in- and outgoing edge weights, respectively. The nodes (emotions) with the highest in- and out-strength were then compared between groups by means of visual inspection. Third, the degree of connectivity was compared between the healthy and MDD groups. This was done by considering network density, which is defined as E/(V*(V– 1)) where *E* is the number of edges and *V* is the number of nodes [33]. Autoregressive effects were not included in this density measure, but a variant that does include autoregressive effects was also computed. Using a Monte Carlo permutation test [34] it was investigated whether the two group networks differed significantly in terms of network density. In this test, the original group members were repeatedly reshuffled (30,000 times) into two new groups, after which the network density was recomputed. Plotting the histogram of the resulting differences indicates how ‘extreme’ the density difference in the original data is compared to what would be expected based on random variation alone. This permutation test was performed in each imputed dataset and the results were averaged.

#### Analyses of the individual networks

Individual networks were estimated using the same procedures as above, but now separately for each individual. After fitting the individual networks, it was investigated if and to what extent variations in network density across individuals were related to having an MDD diagnosis, as previous studies suggested that a depressed state may be characterized by more highly connected emotion networks than a healthy state [15, 17, 18]. In addition, to gain more insight into other possible determinants of inter-individual network differences, the associations between subjects’ network densities and baseline neuroticism, BDI scores, and mean NA levels were analyzed with univariate linear regression analyses. The association between network density and BDI score was analyzed per group, because the BDI score was also used as an inclusion criterion, leading to a clear design-based distinction in BDI sum score between the groups. Regression models with NA and neuroticism, respectively, as the independent variables were run in both groups combined. All preprocessing, missing data imputation and statistical analyses were performed in R (version 3.2.0.) All used scripts are available from the first author.

## Results

### Sample descriptive information

Demographic information about the MDD and healthy groups is shown in Table 1. The groups did not differ on any of the baseline characteristics except the BDI score and medication use. The mean level and standard deviations of each item per group is shown in the supplementary S1 Table. The healthy group scored significantly lower on the negative emotions and higher on the positive emotions compared to the depressed group. Within-person standard deviations of almost all negative emotions were significantly smaller in the healthy group compared to the depressed group (see S2 Table). As regards the positive emotions only one item had a significantly smaller within-person standard deviation in the healthy group.

**Table 1:** Demographic and clinical characteristics of MOOVD participants.

	Depressed (n = 27)	Non-depressed (n = 27)
Age, years (sd)	34.7 (9.9)	34.0 (9.0)
Female, n	20	20
BMI, kg/m^2^ (sd)	24.2 (6.0)	22.5 (2.6)
Smoker, n	7	6
Level of education, n		
Low	0	0
Middle	14	13
High	11	13
Missing	2	1
Employment, n		
Employed	8	14
Student	6	8
Unemployed	8	3
Other	4	2
Missing	1	0
BDI score (baseline) (sd)	31.3 (10.0)	2.3 (2.7)
Medication use, n	17	3
Antidepressants use, n	14	1

Note: BMI = Body Mass Index, BDI = Beck Depression Index.

### Population networks

**Fig 1:**
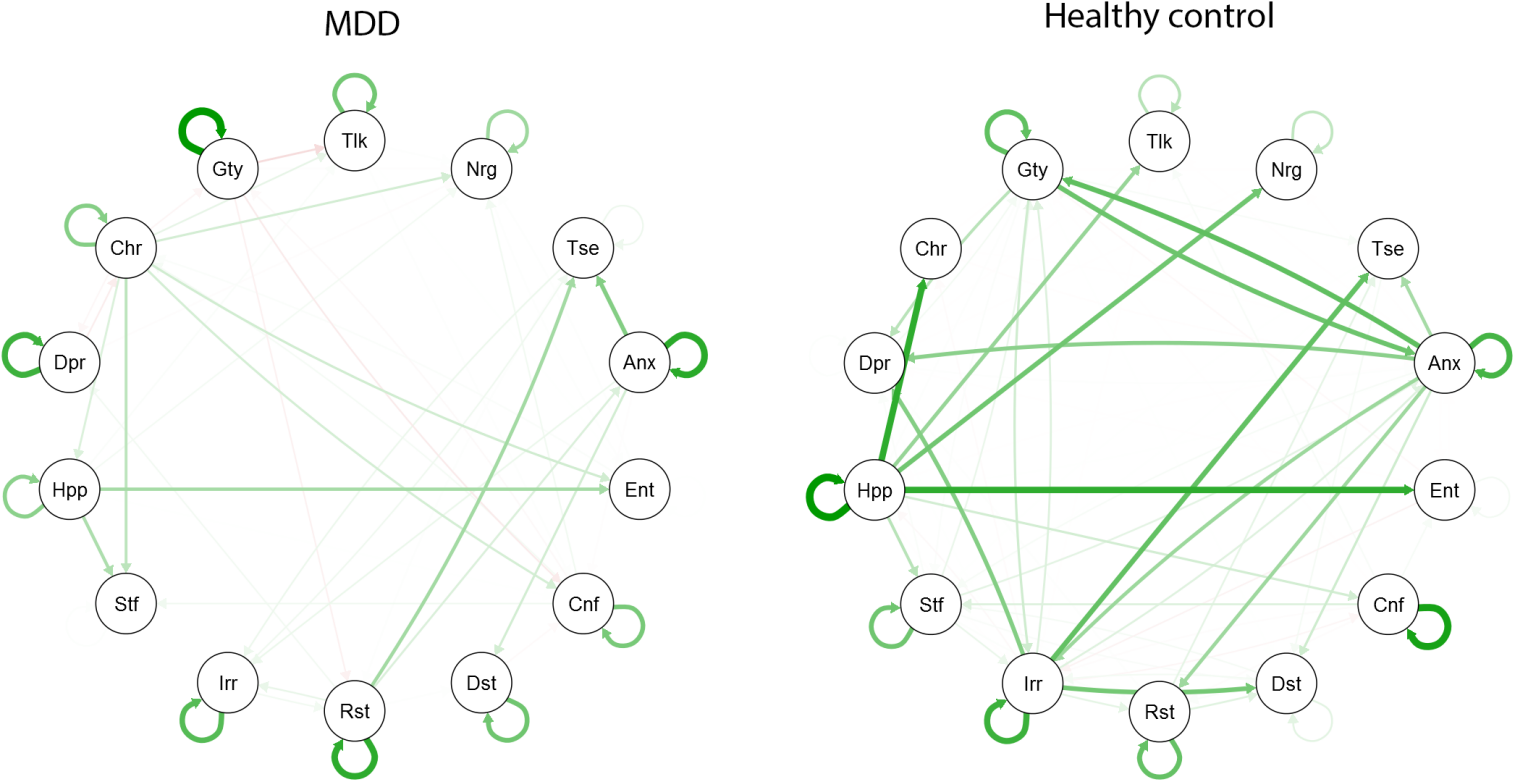
Population networks for the MDD group (left) and control group (right). The networks show longitudinal associations between 14 emotion items. Green and red arrows correspond to positive and negative regression coefficients, respectively. An arrow being more opaque means a stronger connection, i.e. representing a larger regression coefficient.

The results of the sparse VAR analyses in the MDD and healthy groups are shown as longitudinal networks in Fig 1. The MDD group network was characterized by a lower mean in- and out-strength across nodes than the control group network (see Table 2) and also showed less variability in in- and out-strength across nodes (lower standard deviations).

**Table 2:** Mean in- and out-strength for the population networks.

	MDD network		Control network	
	In-strength	Out-strength	In-strength	Out-strength
Mean	0.11	0.11	0.27	0.27
Standard deviation	0.07	0.12	0.12	0.32
Median	0.09	0.07	0.24	0.13
Min	0.03	0.00	0.09	0.00
Max	0.28	0.41	0.47	0.80

**Fig 2:**
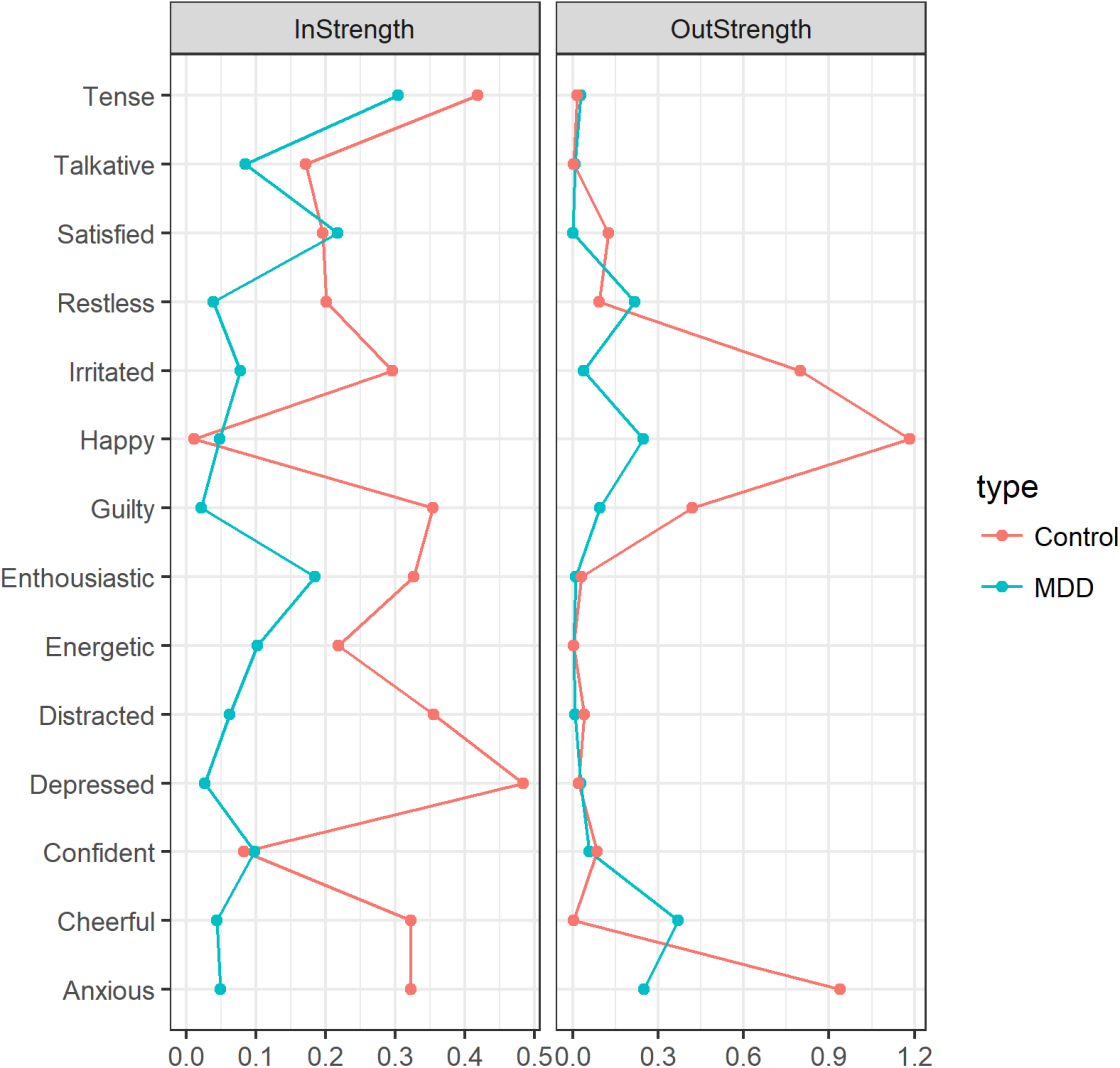
Item-specific in- and out-strengths for the population networks in the MDD and control groups. X-axis values indicate the mean item-specific in-strength and out-strength for the MDD group (blue) and the control group (red).

The item-specific in- and out-strengths for both population networks are shown in Fig 2. Inspection of Fig 2 shows that, overall, the in- and out-strength were larger for the control network nodes. The four nodes that had the highest out-strength in the control network were 'Anxious', 'Irritated', 'Guilty' and 'Happy'. In the MDD group, the four nodes with the highest out-strength were 'Cheerful', 'Restless', 'Happy' and 'Anxious'. The four affective items with the highest in-strength in the MDD network were 'Enthusiastic', Confident’, ‘Tense’ and 'Satisfied'. In the control network, 'Tense', and 'Depressed' had the highest in-strength. Taken together, these results show that the nodes in the control network had a stronger average temporal association with their neighboring nodes compared to the nodes in the MDD network.

### Comparing network density across groups

The difference in network density between the two groups in the original data configuration was -0.12 (MDD group: 0.28, healthy group: 0.40), meaning that the network density was higher in the healthy group than in the MDD group (see Table 3). Averaging over the imputed datasets, the graph density difference was found to be in the bottom 3% of all graph density differences found in the permutation test. This indicated that the observed difference in density between the networks of the MDD and healthy groups was statistically significant. When autoregressive effects were included, the densities were 0.28 for the MDD group and 0.38 for the healthy group. The undirected networks of partial correlations between the residuals of the VAR models, reflecting the contemporaneous associations between the emotions, are shown in S1 Fig. Inspection of these figures shows that there was a higher number of connections in the MDD group than in the healthy control group.

**Table 3:** Population-level density values for the MDD and Control group networks, from different model procedures.

Method	Preprocessing	Density definition	MDD	Control
***Original analyses***					
Sparse VAR	Detrending + Transformation (group-wise)	#Edges (without ar)	0.28	**0.40**
Sparse VAR	Detrending + Transformation (group-wise)	#Edges (including ar)	0.28	**0.38**
Sparse VAR	Detrending + Transformation (group-wise)	Average	0.02	**0.03**
***Comparison with the multilevel approach***					
Multilevel	Person-mean centering	#Edges	**0.21**	0.15
Multilevel	Person-mean centering	Average	**0.04**	0.03
Multilevel	Person-mean centering + Night lag excluded	#Edges	**0.15**	0.10
Multilevel	Person-mean centering + Night lag excluded	Average	**0.02**	0.01
***Multilevel with our preprocessing steps***					
Multilevel	Detrending + Transformation (group-wise)	#Edges	0.24	**0.31**
Multilevel	Detrending + Transformation (group-wise)	Average	0.05	0.05
Multilevel	Detrending + Transformation (per individual)	#Edges	**0.14**	0.11
Multilevel	Detrending + Transformation (per individual)	Average	0.03	0.03

Note. Sparse VAR: Sparse vector autoregressive approach as described by Abegaz and Wit, 2013.

Multilevel: Univariate multilevel regression approach as described by Bringmann et al., 2013.

Detrending: subtracting a smoothing spline from each series.

Transformation: normal quantile transformation as described in Bogner et al., 2012.

Detrending and transformation were done on imputed series, because of estimation difficulties with missing data.

#Edges in sparse VAR approach: network density = (# remaining edges)/(# possible edges)

#Edges in multilevel approach: network density = (# significant edges)/(# possible edges)

Average density in sparse VAR approach: network density = average of absolute edge weights of remaining edges.

Average density in multilevel approach: network density = average of all absolute edge weights.

All density measures include autoregressive (ar) effects, unless otherwise indicated.

In bold: highest densities of the two groups, for ease of comparison.

### Individual networks

**Fig 3:**
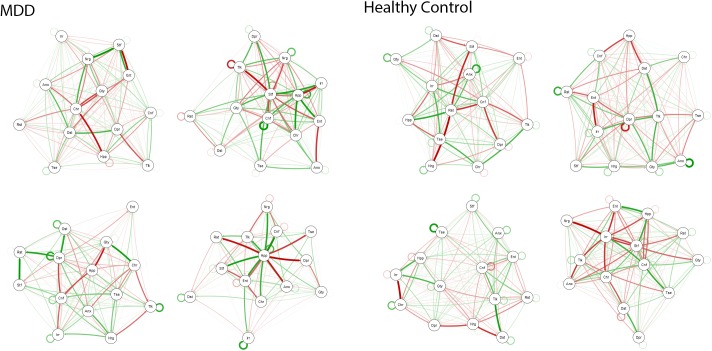
Individual networks for MDD patients and controls. Networks of 4 individuals from the MDD group (left) and 4 individuals from the control group (right). Tlk = Feeling talkative; Nrg = Feeling energetic; Tse = Feeling tense; Anx = Feeling anxious; Ent = Feeling enthusiastic; Cnf = Feeling confident; Dst = Feeling distracted; Rst = Feeling restless; Irr = Feeling irritated; Stf = Feeling satisfied; Hpp = Feeling happy; Dpr = Feeling depressed; Chr = Feeling cheerful; Glt = Feeling guilty.

In Fig 3, within-individual longitudinal networks are shown for 4 randomly selected persons from each group. Visual inspection of the network figures revealed strong heterogeneity in network characteristics across subjects. Next, the mean in-strength and out-strength were calculated for each subject’s network nodes. In the complete sample, the mean in-strength ranged from 1.13 to 1.97 and the mean out-strength ranged from 1.21 to 2.85 across subjects. In the MDD group, the mean in-strength ranged from 1.14 to 1.97 and the mean out-strength ranged from 1.21 to 2.85. In the healthy group, the mean in-strength ranged from 1.13 to 1.81 and the mean out-strength ranged from 1.21 to 2.11. This suggested that network connectivity differed strongly across individuals and that these differences were not explained by top-down defined diagnostic group (MDD or control); regressing graph density on the group label revealed no statistically significant difference between the groups (with an estimate of -0.005, s.e. = 0.023, p = 0.81). In- and out-strength of individual networks was on average four times higher compared to the population networks.

### Determinants of network density

In the pooled data, univariate regression analyses with each individual's graph density as dependent variable and neuroticism as independent variable showed no significant associations (coefficient = -0.003, s.e. = 0.009, p = 0.30, 95% CI = -0.009 to 0.002). Also, NA score was not significantly associated with network density (coefficient = 0.003, s.e. = 0.003, p = 0.30, 95% CI = -0.002 to 0.01). The association between baseline BDI score and graph density was analyzed in the two groups separately, and was non-significant in both cases (in the MDD group: coefficient = -0.0006, s.e. = 0.002, p = 0.743, 95% CI = -0.004 to 0.003; in the control group: coefficient = 0.002, s.e. = 0.006, p = 0.723, 95% CI = -0.004 to 0.003).

### Comparison with the multilevel approach to dynamic networks

The population-level results of this paper are in striking contrast with previously published work on this topic [15, 17, 18], which could be due to the different analytic approach. We addressed this by comparing the results of the abovementioned population-level analysis with results from a multilevel approach as used in these previous papers and described by Bringmann et al. [16]. Previous studies using the multilevel approach also used a different definition of network density than the one used in the current paper, namely, the average absolute edge strength of the network (using all edges, also the non-significant ones). Applying this density definition to the results from the multilevel models, we obtained a graph density of 0.04 for the MDD network and 0.03 for the control network. Applying our own definition of network density (the number of edges in the network divided by the number of possible edges) to the multilevel data, we obtained a graph density of 0.21 for the MDD network and 0.15 for the control network (see Table 3). So, both density measures indicated that the MDD network was denser than the control network when we used the multilevel approach. Applying Bringmann’s definition of network density to the networks obtained from our sparse approach, we got a lower density for the MDD network (0.02 for MDD vs 0.03 for control), in line with our original results (#edges: 0.28 for MDD and 0.38 for control). So, both density measures indicated that the control network was denser than the MDD network if we used the sparse VAR approach. When we used our preprocessing method together with the multilevel approach, the network density was also higher in the control group (#edges: 0.24 for MDD and 0.31 for control); while average densities were equal (0.05 and 0.05, respectively). This suggests that the discrepancy in the results from the two approaches was due to differences in preprocessing rather than to differences in modeling strategy. Further analyses showed that an important part of the discrepancy could be explained by the fact that we preprocessed the data at the level of the groups in our population-level analyses. When we applied our preprocessing method at the level of the individual and used the multilevel approach, a slightly higher density in the MDD group (#edges: 0.14 for MDD and 0.11 for control) or equal densities (average: 0.03 for MDD and 0.03 for control) were found, depending on the density definition. This suggests that our normal quantile transformation and detrending strategy also explained part of the discrepancy.

### The effect dropping the overnight lags

The multilevel analyses were also run with the overnight lag set to missing (see Table 3), using person-mean centering as preprocessing step. The density measures were lower than for the networks estimated in the full data, with higher density in the control group than in the MDD network.

## Discussion

The current study used a VAR-based regularized network method to enable an investigation of how emotions are associated with each other over time within persons. Population-level analyses indicated that the temporal emotion network of the healthy group was significantly more strongly connected (higher density) than the network of the MDD group. This difference was also evident from the mean in- and out-strengths that were calculated for both population networks: nodes in the healthy group networks had stronger temporal associations with their neighbors than their counterparts in the MDD group network. The individual-level network analyses suggested that there was a very high level of heterogeneity across subjects in the characteristics of their networks. Importantly, variability in individual network density was not explained by group membership (MDD vs. healthy). Moreover, network density was not associated with baseline BDI, NA or neuroticism scores. Furthermore, another difference between the individual and population networks was the fact that in- and out-strength tended to be on average four times larger in the individual network. The reason for this finding is as of yet unknown to us. Interestingly, the sparse VAR-based results differed from results obtained with the multilevel approach that has been used previously to estimate longitudinal networks [16]. Networks based on the latter approach showed higher density in the MDD group than in the healthy group. Furthermore, estimated network densities differed considerably depending on the used preprocessing steps. These results are discussed in more detail below.

The results of the current study have important methodological implications. First, the apparent discrepancy between the population-level and individual-level results implies that at least one of the two is not accurate. The used sparse VAR approach is a constant coefficient approach, which relies on the assumption that individuals are similar to each other as regards the studied parameters, but the individual-level analyses suggest the opposite. The individual-level results may reflect true heterogeneity, but may also reflect noise. It has been suggested that a unit-by-unit approach is a preferable when between-subjects heterogeneity is large and the number of time points is high, but that a population-level approach (with either constant, fixed or random effects) is more accurate in case of homogeneity and short or noisy time series [32], but it is hard to tell whether there is true heterogeneity or not, and whether fluctuations are real variations or just noise. However, based on the fact that individual differences are ubiquitous in the literature, we are inclined to think that the constant coefficient approach of the sparse VAR approach is too limited.

The question may then arise why we did not simply use a random effects multilevel approach, as was done in previous studies on dynamic networks [15–18]. The main reason was that the sparse VAR approach allows for estimation of networks with multiple nodes. In a multilevel model with random effect variances and covariances for all parameters, the number of parameters grows rapidly with increasing number of nodes, leading to estimation problems. Indeed, when we tried to apply the multilevel approach to our data we had to use a very parsimonious random effect structure to get the model running at all. Another advantage of the sparse VAR approach is that it is a vector model, meaning that dependencies in the error structure due to contemporaneous associations across the variables can be estimated directly during the estimation process. The multilevel approach consists of separate univariate regression equations, which implies that the dependencies between the error terms of the different equations cannot be estimated directly. This potentially leads to bias in the estimated coefficients (see also the appendix of [16]). Our results showed that the contemporaneous associations were many, especially in the MDD group, so it is not unlikely that the fact that these are not directly estimated in the multilevel models will bias the results. Nevertheless, univariate regression equations can, under certain circumstances, produce a network with a correct structure, even though the regression weights will be biased [35]. This would imply that, when investigating the characteristics of a network derived from multilevel analyses, it is safer to focus on the absence/presence of edges rather than the edge weights (see also below).

The obvious advantage of the multilevel approach is that random effects can be included in the model, so potential heterogeneity in the effects can be accounted for. Population-level findings from our sparse VAR approach were in striking contrast with previous findings from a multilevel approach [15, 17, 18]. These previous studies reported that higher connectivity in emotion networks was related to higher disorder severity, and this was explained with the notion that higher connectivity leads to positive feedback loops and thus increasing and/or persisting emotions. The sparse VAR results did not align with this theory: network connectivity was higher in the healthy group and no associations between the individual network densities and severity indicators were found. When we repeated our analysis using the multilevel approach, this yielded opposite results: now network density was higher in the depressed group, replicating the previous multilevel studies.

Thus, it seems that methodology plays a big role in determining what kind of conclusion we draw about the density of population networks. Applied to the same data, the two approaches yielded opposite conclusions. Our additional analyses showed that the discrepancy in the results was most likely due to differences in preprocessing methods, rather than to differences in modeling strategy. Higher density in the control group was also found with a multilevel approach, when we used our original preprocessing method. However, applying our preprocessing method at the level of the individual instead of pooled over individuals of the groups, the multilevel approach showed an equal density or a slightly higher density in the MDD group, depending on the density definition. This shows the importance of preprocessing at the proper level when groups are heterogeneous, and thus should probably be seen as a further invalidation of our population-level results. Other aspects of our preprocessing also seemed to explain some part of the discrepancy, for example whether or not the data were transformed. Some previous studies applied linear regression techniques to variables that were probably rather skewed. Especially in ESM data of healthy participants, floor and ceiling effects are often present. This may bias the results; a recent study showed that the higher density in the MDD group reported in a previous study was eliminated if a generalized approach was used to deal with the skewness of the data [36]. Our variables were also skewed, especially the negative items in the healthy group. Our normal quantile transformation was an attempt at alleviating this problem. Indeed, the multilevel models without this transformation step returned skewed distributions for the residuals of several of the emotions, especially in the healthy group. The distribution of the residuals of the sparse VAR models better resembled the normal distribution, although distributions were still not optimal for some items. Future work may help to find still better ways to deal with non-normal data. Another preprocessing aspect is if and how the data are detrended. We subtracted a smoothing spline from each of the series to ensure stationarity, whereas in the multilevel approach, models with and without a trend variable were compared using the BIC. All in all, it seems that more work is needed to determine the optimal way of preprocessing network data, especially when they are skewed. Until that time, inferences about the link between increased emotion-network connectivity and increased severity should be made with caution, as the methods used have varied considerably and appear to have great influence on the outcomes.

In this study also a different definition of graph density was used. Previous studies used the average of the absolute values of all edges, whereas we used the number of remaining edges in the penalized models divided by the number of possible edges. This difference, however, did not explain the discrepancy in the results from the sparse VAR approach and the multilevel approach, although the values were of a different order of magnitude. We chose the latter definition because it is a common definition of graph density in a directed network in the field of applied mathematics (e.g., [33]). Obviously this does not make our choice of density measure correct in itself and we think it is still a matter of debate which definition of density is the most appropriate for our field. We think both definitions have their advantages and disadvantages. Averaging the absolute values has the disadvantage that also non-significant values are included while such edges may merely represent noise/spurious associations. Averaging only the significant values would resolve that problem but would lead to other peculiarities: if only one edge with a large estimate would be significant, average density would be very large. Our own density measure has the advantage that non-significant (and thus potential spurious) associations are not included, but has the disadvantage that the size of the associations is not accounted for. Moreover, the penalized approach may eliminate many small-sized edges, which may be relevant from a substantive point of view. It is still unclear what is most important as regards density: the number of connected nodes, the size of the connections, or both. One could argue that using a density measure based on the number of edges is less affected by biased coefficient estimations. Therefore, from a pragmatic perspective, using such a density measure could be particularly useful when a network is estimated based on the multilevel approach, which can have biased edge weights, as discussed above.

The obvious problem of our sparse approach is that it is a constant coefficient model, in which homogeneity in the effects across individuals is assumed. This is probably not very realistic, as also our individual-level analyses suggest. The possibility to include random effects to account for such heterogeneity is the great advantage of the multilevel approach. The problem with the multilevel approach, however, is that models become easily overparameterized with higher number of nodes. Thus, it seems that a combination of the two approaches is needed: an approach that includes both a regularization method and a method to account for heterogeneity in the effects. A Bayesian approach, as has recently been advanced [37] may also be a fruitful road to explore. Hopefully, future research can yield such improved methods.

The current study also illustrated the possibility to generate longitudinal emotion networks for individual subjects using VAR-model estimations. Because the sparse VAR model uses regularization, this approach can also work with short time series (i.e. little data per model parameter) and many nodes. The networks varied strongly across individuals. If these results truly reflect reality, this has clear implications for the generalizability of population-level networks to individuals within these populations. For instance, based on the population-level networks in this study, one could have expected the healthy group members to show more highly connected individual networks than the MDD group members (or vice versa, when a multilevel approach was used). However, no such relationship between individual network characteristics and group membership was seen. Thus, generalizability of population-level network models to individuals is likely to be limited [38]. From an application perspective, the possibility to correctly estimate n = 1 networks opens up a whole range of new possibilities. In research, estimation of individual networks allows network characteristics (e.g. density, mean in/out-strength) to be treated as subjects’ attributes that vary within the sample and to be used as variables in (multivariate) analyses. In applied settings, well-estimated, empirically-based network models could be of particular interest to those working in e-health and precision medicine. For instance, the method could be used to estimate a personalized network model based on diary data collected by a patient using a smartphone app (or similar device). Such a network can generate feedback for the patient about the influences that particular emotions/feelings have on each other in his or her daily life. Some preliminary work has already been done on such automated feedback systems [39, 40] and the presented methods could help to develop these systems further.

The focus of this study was on the longitudinal (time-lagged) associations between emotions. The advantage of the VAR approach is that it is possible to estimate networks that purely reflect the dynamic associations between emotions over time. By capturing all contemporaneous covariances between the emotions in the error covariance matrix of the VAR model, observed longitudinal associations between t and t-1 that are actually explained by contemporaneous covariances between emotions at t-1 and autocorrelations between t and t-1 are eliminated from the longitudinal network. This is an important feature of the VAR model as it helps to better distinguish the potential order of effect in the relationships between emotions [41]. Nevertheless, it can be argued that these models are incomplete. Often, contemporaneous associations between mental states assessed with diary methods are substantial and this was also the case in our study, especially in the MDD group. The contemporaneous associations reflect to what extent emotions tend to be experienced together, which may be due to shared measurement variance, a common cause, or interactions among the emotions at shorter time intervals. There are approaches in which the contemporaneous associations are included in the model, e.g. structural VAR models [42] and uSEM models [43], but these models cannot be identified unless some a priori restrictions are imposed [41]. If there is a strong theory guiding choices about the ordering of the contemporaneous associations, these approaches may be preferable.

### Limitations

Although the current study had several strengths, including the penalized approach, the considerable length of the time series, the efforts taken to appropriately handle non-normality and missing data, and the possibility to relate network characteristics to patient characteristics, the results should also be interpreted in the light of several limitations. First, the emotion items were scored on a 7-point scale but the used model could only treat them as continuous, which could have influenced the results to some degree. Second, only VAR models with time lag 1 were estimated to keep the models as parsimonious as possible. Although the method can also be used with time lag 2, this would require estimation of numerous additional parameters. Third, the power to detect meaningful associations between the individual network densities and severity measures may have been limited due to the rather small sample size. Fourth, the items used to assess PA and NA are not necessarily comparable to depression symptoms, because symptoms, as per definition, should persist for more than two weeks, and we did not assess problems like suicidality, eating problems and sleeping problems, which may play an important role in MDD. Fifth, the interval between the evening and morning measurements was longer than the other measurements, which may have introduced additional noise in the data. Sixth, the coefficients used are raw coefficients. It has been shown that standardizing coefficients can yield different results [44]. Despite these limitations the current study provided interesting new starting points for further research in this direction, especially as regards methodology.

### Conclusions

The current study presented a VAR-based method for the estimation of sparse population-level and individual-level networks of emotion dynamics over time. The population-level sparse VAR results were in sharp contrast with results obtained from a multilevel approach. Further, population-level results from both the sparse VAR and the multilevel approach were in contrast with individual-level results: whereas the population-based analyses indicated that the within-person emotion dynamics were stronger (sparse VAR) or less strong (multilevel) in healthy subjects than in MDD patients, the individually estimated emotion networks varied strongly across persons and showed no association with diagnostic group, severity and personality. This suggests that either the individual networks are unreliable, or that emotion dynamics are highly personal and not related to top-down defined disorder categories and/or severity constructs. The main conclusion of the study may be that drawing inferences from dynamic emotion networks should be done with great caution until some consensus is obtained as regards the preferred methodology.

## Supporting information

S1 Fig The contemporaneous networks (based on partial correlations of the residuals from the sparse VAR models) for the MDD group (left) and the healthy control group (right). (TIF)

S1 Table Population mean levels and standard deviations for each of the 14 emotion items, per group. (DOCX)

S2 Table Within-person means and standard deviations for each of the 14 emotion items, per group. (DOCX)
