# Supplementary figures and images for: An investigation of emotion dynamics in major depressive disorder patients and healthy persons using sparse longitudinal networks
Source: PLoS One. 2017 Jun 1;12(6):e0178586. doi: 10.1371/journal.pone.0178586 (PMC5453553; doi:10.1371/journal.pone.0178586)

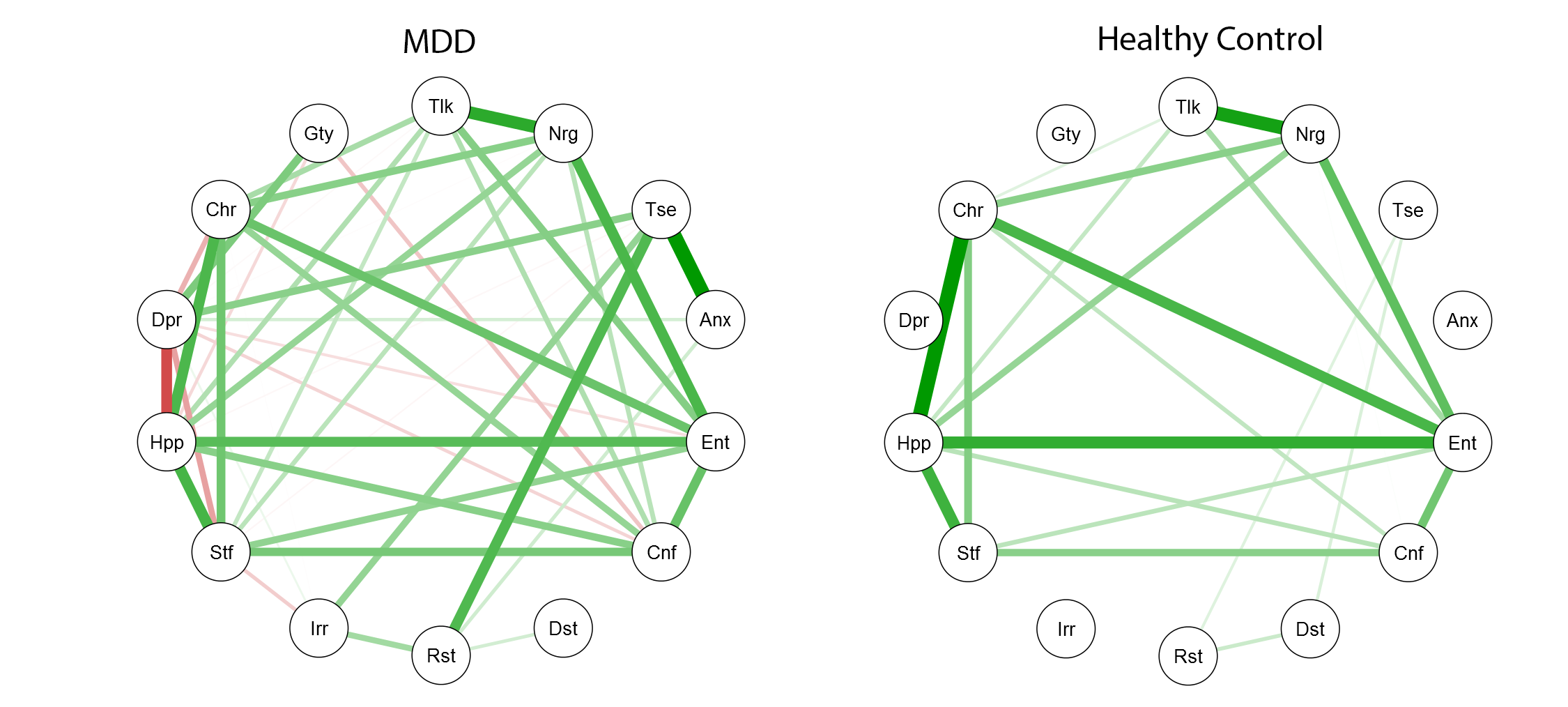

Supplement: S1 Fig — (TIF) [file pone.0178586.s001.tif]
