# Supplementary material for: An investigation of emotion dynamics in major depressive disorder patients and healthy persons using sparse longitudinal networks
Source: PLoS One. 2017 Jun 1;12(6):e0178586. doi: 10.1371/journal.pone.0178586 (PMC5453553; doi:10.1371/journal.pone.0178586)
Supplement: S1 Table — (DOCX) [file pone.0178586.s002.docx]

**S1 Table 1. Population mean levels and standard deviations for each of the 14 emotion items, per group**

|  | MDD  (N=27, #obs=2234)) | | Control  (N=27, #obs=2264) | | T-test for difference in mean | |
| --- | --- | --- | --- | --- | --- | --- |
| Item | **Sample mean** | **Sample**  **SD** | **Sample mean** | **Sample**  **SD** | **t-statistic** | **p-value** |
| Feeling talkative | 3.38 | 1.45 | 4.24 | 1.30 | 20.9 | <.001 |
| Feeling energetic | 3.39 | 1.45 | 4.45 | 1.26 | 26.2 | <.001 |
| Feeling tense | 3.21 | 1.59 | 1.77 | 1.26 | -33.7 | <.001 |
| Feeling anxious | 2.51 | 1.52 | 1.14 | 0.52 | -40.5 | <.001 |
| Feeling enthusiastic | 3.37 | 1.43 | 4.50 | 1.30 | 27.7 | <.001 |
| Feeling confident | 3.48 | 1.38 | 4.96 | 1.31 | 37.0 | <.001 |
| Feeling distracted | 3.14 | 1.49 | 1.97 | 1.36 | -27.5 | <.001 |
| Feeling restless | 3.36 | 1.59 | 1.63 | 1.16 | -41.4 | <.001 |
| Feeling irritated | 2.91 | 1.63 | 1.42 | 0.98 | -37.1 | <.001 |
| Feeling satisfied | 3.62 | 1.49 | 4.87 | 1.28 | 30.0 | <.001 |
| Feeling happy | 3.42 | 1.47 | 4.69 | 1.28 | 30.9 | <.001 |
| Feeling depressed | 3.52 | 1.83 | 1.29 | 0.75 | -53.4 | <.001 |
| Feeling cheerful | 3.40 | 1.43 | 4.56 | 1.35 | 28.1 | <.001 |
| Feeling guilty | 2.79 | 1.78 | 1.12 | 0.46 | -42.9 | <.001 |

Note. #obs= number of observations. The MDD group scored significantly higher on the negative emotion items and lower on the positive emotion items (independent sample t-tests)
