# Supplementary material for: An investigation of emotion dynamics in major depressive disorder patients and healthy persons using sparse longitudinal networks
Source: PLoS One. 2017 Jun 1;12(6):e0178586. doi: 10.1371/journal.pone.0178586 (PMC5453553; doi:10.1371/journal.pone.0178586)
Supplement: S2 Table — (DOCX) [file pone.0178586.s003.docx]

**S2 Table. Within-person means and standard deviations for each of the 14 emotion items, per group**

|  | MDD  (N=27) | | Control  (N=27) | | T-test for difference in mean | | T-test for difference in SD | |
| --- | --- | --- | --- | --- | --- | --- | --- | --- |
| Item | **Within-person mean** | **Within-person**  **SD** | **Within-person mean** | **Within-person**  **SD** | **t-statistic** | **p-value** | **t-statistic** | **p-value** |
| Feeling talkative | 3.38 | 1.03 | 4.24 | 1.01 | 3.6 | <.001 | -0.3 | .78 |
| Feeling energetic | 3.39 | 1.02 | 4.45 | 1.00 | 4.4 | <.001 | -0.4 | .73 |
| Feeling tense | 3.21 | 1.14 | 1.77 | 0.88 | -5.5 | <.001 | -2.5 | .02 |
| Feeling anxious | 2.51 | 1.00 | 1.14 | 0.33 | -6.3 | <.001 | -6.8 | <.001 |
| Feeling enthusiastic | 3.37 | 0.94 | 4.50 | 0.87 | 4.2 | <.001 | -0.9 | .40 |
| Feeling confident | 3.48 | 0.82 | 4.96 | 0.66 | 5.0 | <.001 | -2.2 | .04 |
| Feeling distracted | 3.14 | 1.10 | 1.97 | 0.93 | -4.6 | <.001 | -1.5 | .13 |
| Feeling restless | 3.36 | 1.13 | 1.63 | 0.69 | -6.4 | <.001 | -4.1 | <.001 |
| Feeling irritated | 2.91 | 1.13 | 1.42 | 0.67 | -6.0 | <.001 | -4.2 | <.001 |
| Feeling satisfied | 3.62 | 0.94 | 4.87 | 0.81 | 4.4 | <.001 | -1.7 | .10 |
| Feeling happy | 3.42 | 0.89 | 4.69 | 0.80 | 4.4 | <.001 | -1.1 | .28 |
| Feeling depressed | 3.52 | 1.10 | 1.29 | 0.55 | -7.6 | <.001 | -6.1 | <.001 |
| Feeling cheerful | 3.40 | 0.92 | 4.56 | 0.92 | 4.3 | <.001 | -0.1 | .95 |
| Feeling guilty | 2.79 | 0.99 | 1.12 | 0.30 | -5.8 | <.001 | -7.9 | <.001 |

Note. SD=standard deviation. Within-person mean and SDs are averages of the within-person means and within-person SDs across individuals in each group. The MDD group scored significantly higher on the negative emotion items and lower on the positive emotion items. Within-person SDs were significantly higher in the MDD group for all negative items except ‘distracted’. Within-person SDs on the positive items did not differ significantly, except for the item ‘confident’ (independent sample t-tests
